# Using BERT to identify drug-target interactions from whole PubMed

**DOI:** 10.1186/s12859-022-04768-x

**Published:** 2022-06-21

**Authors:** Jehad Aldahdooh, Markus Vähä-Koskela, Jing Tang, Ziaurrehman Tanoli

**Affiliations:** 1 Research Program in Systems Oncology, Faculty of Medicine, University of Helsinki, Helsinki, Finland; 2 Institute for Molecular Medicine Finland, University of Helsinki, Helsinki, Finland; 3 Doctoral Programme in Computer Science, University of Helsinki, Helsinki, Finland; 4 BioICAWtech, Helsinki, Finland

**Keywords:** BERT, Bidirectional encoder representations from transformers, BERT for biomedical data, Drug target interaction prediction, Mining drug target interactions, Biomedical text mining, Bioactivity data, Drug repurposing

## Abstract

**Background:**

Drug-target interactions (DTIs) are critical for drug repurposing and elucidation of drug mechanisms, and are manually curated by large databases, such as ChEMBL, BindingDB, DrugBank and DrugTargetCommons. However, the number of curated articles likely constitutes only a fraction of all the articles that contain experimentally determined DTIs. Finding such articles and extracting the experimental information is a challenging task, and there is a pressing need for systematic approaches to assist the curation of DTIs. To this end, we applied Bidirectional Encoder Representations from Transformers (BERT) to identify such articles. Because DTI data intimately depends on the type of assays used to generate it, we also aimed to incorporate functions to predict the assay format.

**Results:**

Our novel method identified 0.6 million articles (along with drug and protein information) which are not previously included in public DTI databases. Using 10-fold cross-validation, we obtained ~ 99% accuracy for identifying articles containing quantitative drug-target profiles. The F1 micro for the prediction of assay format is 88%, which leaves room for improvement in future studies.

**Conclusion:**

The BERT model in this study is robust and the proposed pipeline can be used to identify previously overlooked articles containing quantitative DTIs. Overall, our method provides a significant advancement in machine-assisted DTI extraction and curation. We expect it to be a useful addition to drug mechanism discovery and repurposing.

**Supplementary Information:**

The online version contains supplementary material available at 10.1186/s12859-022-04768-x.

## Introduction

The average cost of developing a new drug ranges in billions of dollars, and it takes 9–15 years to bring a new drug to the market [1]. Hence, finding new uses for already approved drugs is of major interest to the pharmaceutical industry. This practice, termed drug repositioning or drug repurposing, is attractive because of its potential to speed up drug development, reduce costs, and provide treatments for unmet medical needs [2]. Central to drug discovery and repositioning are drug-target interactions (DTI), meaning the qualitative, quantitative, and relative interactions of drugs with the molecules that regulate cellular functions.

DTIs are catalogued in public databases, which classify DTIs as binary (contains both active and inactive interactions), unary (only active interactions) or as quantitative (in terms of IC50, Kd, Ki etc.) [3]. The most well-known databases for quantitative bioactivity interactions are ChEMBL [4], BindingDB [5], PubChem [6], GtopDB [7] and DrugTargetCommons [8], 8]. These resources contain experimental data for millions of compounds across thousands of protein targets. The quantitative DTI data in these databases is manually extracted from experimental articles. None of these drug-target databases provide target coverage for approved drugs at the whole proteome level, and only 11% of the human proteome are targeted by small molecules [10]. The combined non-overlapping articles covered by these five databases numbered less than 0.1 million, and contain around 3,000 protein targets with an average of 7.33 interactions per target [11].

To overcome the limited coverage of DTI profiles in the public databases, several in-silico DTI prediction studies are proposed. For instance, the IDG-DREAM Challenge is based on crowdsourcing-based AI and ML methods to predict target activities for kinase inhibitors [12]. Thafar et al., predicted new DTIs using graph embedding and similarity based approaches [13]. Similarly, Zheng et al., used multiple kernels into a tripartite heterogeneous drug–target–disease interaction spaces to predict DTIs [14]. Several other computational approaches have been developed over the past decade, providing systematic means for predicting potential DTIs [15–17]. These in-silico methods provide a deeper understanding of the factors affecting DTI prediction and have opened novel strategies for computational drug repurposing.

Another alternative strategy is the curation of DTIs from experiment-based articles, adapted by several major databases such as ChEMBL, BindingDB and DrugTargetCommons. However, each resource focuses only on specific journals for data curation. For instance, ChEMBL and DrugTargetCommons primarily focus on Medicinal chemistry, Nature biotechnology and a few other journals. However, there are more than 7000 journals and 32 M articles on PubMed [18]. A large fraction of the non-curated articles may contain experimentally tested DTIs. However, curating the whole PubMed manually is not efficient. Therefore, there is a need to develop semi-automated text classifiers to identify the most relevant articles.

Text classification is a well-known problem in natural language processing (NLP). The objective is to assign predefined categories to a given text sequence (in this case, it could be an abstract, title or full text for the article). One of the pre-processing step is to map textual data into numerical features [19], to make it understandable by the prediction model. Mapping of textual information into numerical features can be performed using pre-trained models on a large corpus of texts. Pre-trained language models on large text corpora are proven to be adequate for the task of text classification with a decrease in computational costs at runtime [20]. Among those are the word embedding based models, such as word2vec [21] and GloVe [22], as well as contextualized word embedding models, such as CoVe [23] and ELMo [24]. Others are sentence-level models, such as ULMFiT [25]. More recently, pre-trained language models are shown to be helpful in learning common language representations by utilizing a large amount of un-labelled data, e.g., OpenAI GPT [26] and BERT [27]. Bidirectional Encoder Representations from Transformers (BERT) is based on a multi-layer bidirectional Transformer and is trained on large plain texts for masked word prediction and next sentence prediction tasks.

PubTator [28] and BEST [29] are currently the two most comprehensive web platforms that can automatically mine drug and target proteins from PubMed or PubMed Central (PMC). However, these tools did not capture the DTIs, and the resulting output may or may not contain experimental data. To solve these shortcomings, we set out to construct a pipeline using a BERT-based text classifier to identify articles containing DTIs and extract the associated data from PubTator. We trained several BERT models (i.e., BERT, SciBERT [20], BioBERT [30], BioMed-RoBERTa [31] and BlueBERT [32]) on known articles containing DTIs and used majority voting of five BERT models to predict 0.6 M new articles. The identified articles are further linked with mined drug and protein entities provided by PubTator. Furthermore, the BERT models predicted the assay format used in the experiment with an F1 micro of 88%. The resulting predicted and integrated datasets are freely available at https://dataset.drugtargetcommons.org/. The script for generating these models is freely available at: https://github.com/JehadAldahdooh/DTIs.

## Materials and methods

### Drug and protein annotations for PubMed articles

We downloaded drug and protein annotations for the abstracts of 24 M documents (75% of the PubMed) using PubTator’s API [28]. We define here document as a merged text containing titles and abstracts for the articles. Approximately a quarter of the articles in PubTator missed the abstract information. We considered only those articles for which both abstract and title information is present in PubTator, after which **18.5 M** documents remained.

### Known articles for drug-target bioactivity data

Data used for the model training contains 28,075 positive examples (articles containing drug-target bioactivity data) and 28,075 negative examples (other biological articles), which is available at: https://dataset.drugtargetcommons.org/Training_DTIs_data/. We considered only those articles in the positive dataset that contain both drug and protein annotations in PubTator. Drug-target articles are extracted from DrugTargetCommons and ChEMBL (27th release), whereas data for other biological documents is extracted from DisGeNET [33]. We used DisGeNET as a negative dataset mainly because it is a comprehensive and manually curated database for disease and gene associations. Trained models are then used to predict documents that are likely to contain DTIs. Finally, the predicted documents likely to contain DTIs are associated with drug and protein entities as identified by PubTator.

### Assay formats for drug-target bioactivity data

Furthermore, we trained our models to predict the assay format most likely used in the documents. Assay format annotations are extracted from DrugTargetCommons for 28,102 documents with 14,109 focusing on cell-based assays, 12,845 having organism based and 1,148 as other assay formats (e.g., biochemical (93), cell-free (66), tissue-based (424) and physiochemical (565)). The training data for assays is available at https://dataset.drugtargetcommons.org/Training_assay_data/.

### Proposed methods

BERT base is a masked language model (MLM) with 12 layers of architecture, pre-trained on > 2.5B words from English Wikipedia. We used BERT base and other BERT models (SciBERT, BioBERT, BioMed-RoBERTa and BlueBERT) to identify new articles on PubMed likely to contain DTIs. SciBERT is an MLM pre-trained model trained on 1.14 M full-texts from Semantic Scholar corpus with 82% from the biomedical domain [34]. SciBERT uses a different vocabulary (SCIVOCAB), whereas BERT, in general, is based on BASEVOCAB. In this study, we adapted uncased SciBERT. BioBERT is an MLM pre-trained language model based on the BERT representation for the biomedical domain. We used BioBERT-v1.1, pre-trained on PubMed for 200 K steps and 270 K steps on PMC. The model is pre-trained using the same hyper-parameter settings as for the original BERT model. BioMed-RoBERTa is a MLM pre-trained language model based on the RoBERTa [31]. Finally, BlueBERT is pre-trained on approximately 4B words extracted from PubMed.

To fit the training data into the BERT models, we preprocessed it by applying the tokenization to break up the text into tokens. We used the class AutoTokenizer from the HuggingFace Transformers package [35]. It allows to instantiate a tokenizer for the selected BERT model and format the text by adding the special [CLS] token at the beginning of each text and [SEP] token at the end of the sentences. It also pads or truncates the resulting vectors to a standardized length limit of BERT model (512 tokens at a time).

We used the BERT representations for the classification task by fine-tuning the BERT variants with minimal changes applied during the training phase. All the BERT models used in this analysis comprised of 12 layers of transformer encoder with hidden state dimensions equal to 768 and having > 110 M parameters as adopted in [36]. In our architecture, we have used the embedding vector of the BERT [CLS] token from the last hidden layer as a representation of each textual sequence. It is further processed by two fully connected layers and a SoftMax activation function.

**Fig. 1:**
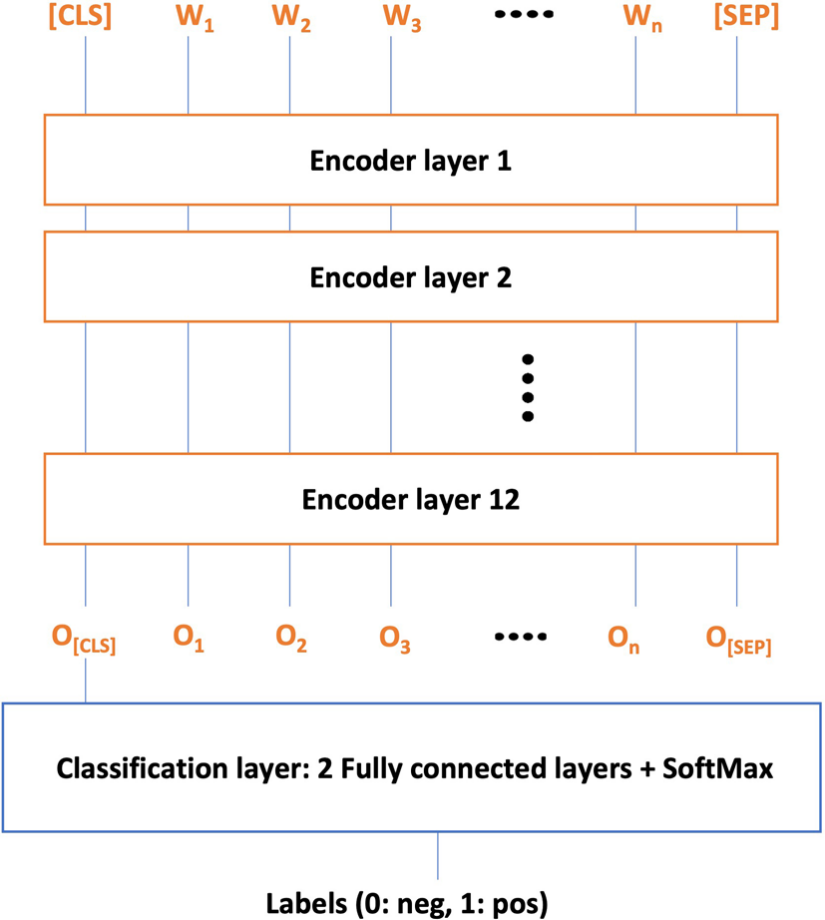
Architecture for all the BERT models, where Wi represents input word token and O_i_ represents contextual embeddings at the output layer. The O_[CLS]_ is first token of output sequence and contains class label

The BERT variants are fine-tuned using NVIDIA Tesla V100 SXM2 32 GB GPU, with a batch size of 32, a maximum sequence length of 512, a learning rate of 2e-5 for DTIs task, 5e-5 for assay classification task, a maximum epoch size of 3 for DTI prediction task, and 9 for assay prediction task. We used Adam with β_1_ = 0.9 and β_2_ = 0.999, slanted triangular learning rates as in [25], warm-up portion to 0.1, and ensured that GPU memory is fully utilized. The model architecture for all the BERT models in this study is shown in Fig. 1.

Next, we divided the overall workflow into three modules:

1. To identify whether a PubMed article is likely to contain bioactivity data for drug-target interactions.
2. To extract drug and protein information by taking advantage of already extracted entities by PubTator.
3. To predict assay format for positively identified articles.

**Fig. 2:**
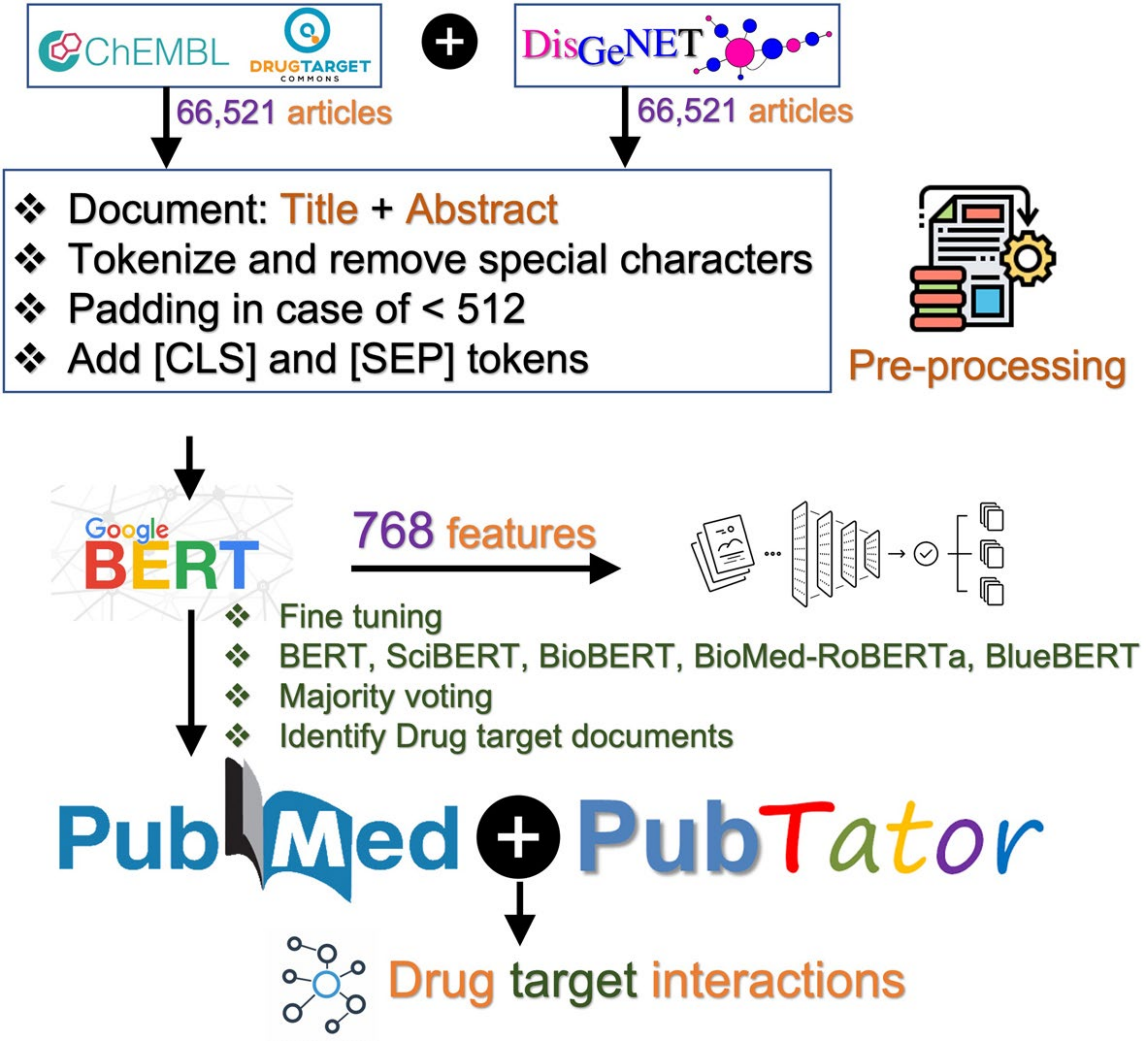
Workflow for identifying new articles containing drug-target bioactivity data

For module 1, we used the fine-tuned BERT models to predict whether PubMed’s article contains a drug-target relationship or not. The BERT models are trained on 28,075 positive and 28,075 negative documents as explained in the previous section. Each document is mapped into 768 numerical features with minor differences in the architecture of the five models. After training, individual BERT models are merged in a majority voting to identify new articles possibly containing DTIs. We used the majority voting because different BERT models performed differently on the external test datasets (Table 2) and the majority voting may reduce the risk of false positives. For module 2, we then matched and linked positively predicted documents with annotated drug and protein entities using the PubTator dataset. Finally, for module 3, using the same model architecture, we tried to predict assay formats (cell-based, organism based or other assays) for the positively predicted documents in module 2. We emphasized on the assay format prediction because assay formats are critical in defining the confidence scores for DTIs [37]. We reported these predicted articles in https://dataset.drugtargetcommons.org/New_predictions/. The workflow of the proposed strategy is shown in Fig. 2.

## Results and discussions

### Ten-fold cross-validation results using BERT models

The BERT text classifiers are trained using tenfold cross-validation**.** Our analyses showed that all the BERT models reached accuracies higher than 99%. Furthermore, we tested BERT models on three independent datasets i.e. DrugProt [38] (a positive dataset), Medline (a negative dataset) used by Papadatos et al., [39], and non-overlapping articles from ChEMBL (a positive dataset). As shown in Table 1, BioBERT achieved an accuracy of 71.5% on the DrugProt dataset, while BlueBERT was able to correctly identify negative articles from Medline with 100% accuracy, and SciBERT successfully identified positive articles from ChEMBL with 93.2% accuracy. Using manual curation, we also validated 100 DTIs articles (from 0.316 M articles at PubTator that are predicted as DrugTarget articles and contain both drug and protein entities). We confirmed that all the articles contain relationship words (such as inhibition or binding) in the abstracts of the articles. These 100 articles are provided in Additional file 1. As the model was primarily trained on the subset of PubTator showing 99% accuracy, that is why we obtained 100% accuracy on those 100 articles. Articles in DrugProt datasets are more complex and are different from PubTator (0.31 M articles). Especially, DrugProt focusses on several types of relationships (including substrate, up regulators, down regulators, and others) which are not in the scope of current study. Therefore, we did not include those types of articles in model training, resulting in slightly reduced accuracy for the DrugProt dataset. However, high performance at Medline and ChEMBL datasets depicts the generalizability of BERT models to identify drug-target like articles with great precision.

**Table 1:** Accuracy of BERT models on three independent datasets. DrugProt is the dataset containing 2788 positive articles based on DTIs (positive class) and 1215 from negative articles class. Medline is a completely negative dataset, and ChEMBL is a completely positive dataset containing DTIs

Dataset	Articles	BERT	SciBERT	BioBERT	BioMed-RoBERTa	BlueBERT	Majority voting
DrugProt	4003	68	65.9	**71.5**	71.4	67.5	69.6
Medline	55,056	99.7	98.6	75.2	99.9	100	100
ChEMBL	876	89.6	93.2	91.2	83.4	88.7	90.3

Bold values indicate the top results for a dataset

**Fig. 3:**
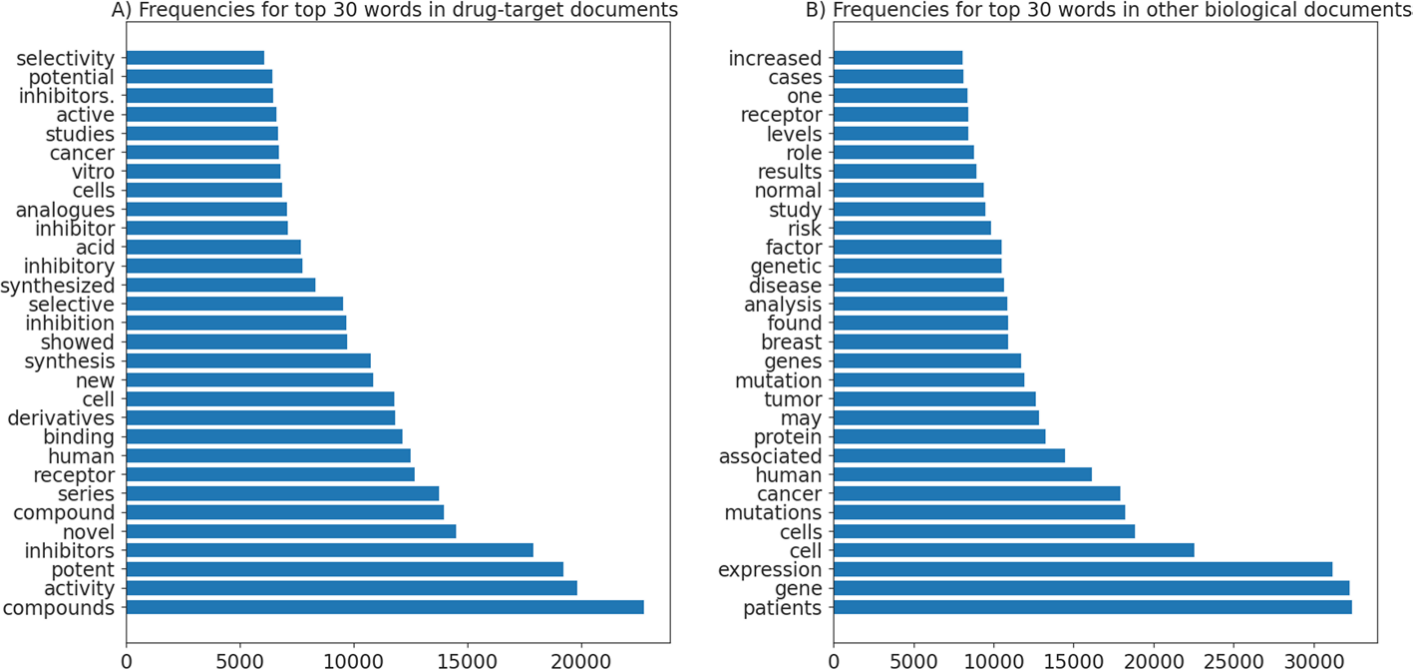
Top word frequencies for **A** Drug-target documents, and **B** Other biological documents

We also compared the top frequently occurring words in both positive and negative documents. As shown in Fig. 3, the most frequently occurring words in drug target documents are ‘compounds’, ‘activity’ and ‘potent’, whereas the most frequent words for other biological documents are ‘patients’, ‘gene’, and ‘expression’. The word distribution analysis can demonstrate developing a simple model based on word frequencies to identify drug-target or other biological documents. Simple model can have good time complexity but at the cost of lower accuracy.

### Identify new drug-target articles and associate drug and protein pairs using PubTator dataset

After successfully training the BERT models, we tried to identify new articles on PubMed that possibly contain bioactivity data for drug-target pairs. For this purpose, we used 18.5 M documents downloaded from PubTator. Table 2 shows the number of positively predicted documents out of these documents. The third column (articles containing drugs or proteins on PubTator) shows how many among positively predicted articles have either drug or protein entities annotated by PubTator. Finally, the last column indicates the number of articles for which PubTator annotated both drug and the protein entities. These two columns validate those articles that are identified as drug-target articles.

**Table 2:** Prediction of drug-target like documents from PubMed articles. The third column shows the number of documents that contain either drug or protein entities as identified by PubTator. In contrast, the fourth column indicates the number of documents that contain both drug and protein entities

BERT model	Predicted as drug-target articles	Articles containing drugs or proteins on PubTator	Articles containing both drugs and proteins on PubTator
BERT	688,206	682,150	342,902
SciBERT	594,999	589,999	321,831
BioBERT	636,091	630,132	340,638
BioMed-RoBERTa	725,748	720,030	**385,015**
BlueBERT	570,284	564,220	297,834
Majority voting	597,844	592,789	316,794

Bold value indicates the top result for a dataset

We obtained a superior performance on unseen articles. For example, using the majority voting, 99% (597,844) of the articles were identified as drug-target like (positive) containing either drugs or proteins entity identified by PubTator. Out of these positively predicted documents, 53% (316,794) contain both drug and protein entities according to PubTator. The result (53%) is likely an underestimation, as drug and protein entities may not appear in the main text of an article but may be deposited as supplementary data, which are not captured by PubTator’s back-end algorithm. It is also possible that drug, and protein entities are present in the main text but were not captured by PubTator. This means that even though the article is positively predicted by our model, we might not be able to capture drug or protein entities in some cases, leaving the task for manual curators to check the supplementary material. Indeed, many high throughput drug-target profiling articles do not mention drug or protein names in the main text but instead provide these in the supplementary material, e.g. [40]. Of the BERT models, BioMed-RoBERTa identified more drug-target like documents compared to the other models, with at least 385,015 articles containing both drugs and proteins in PubTator.

**Fig. 4:**
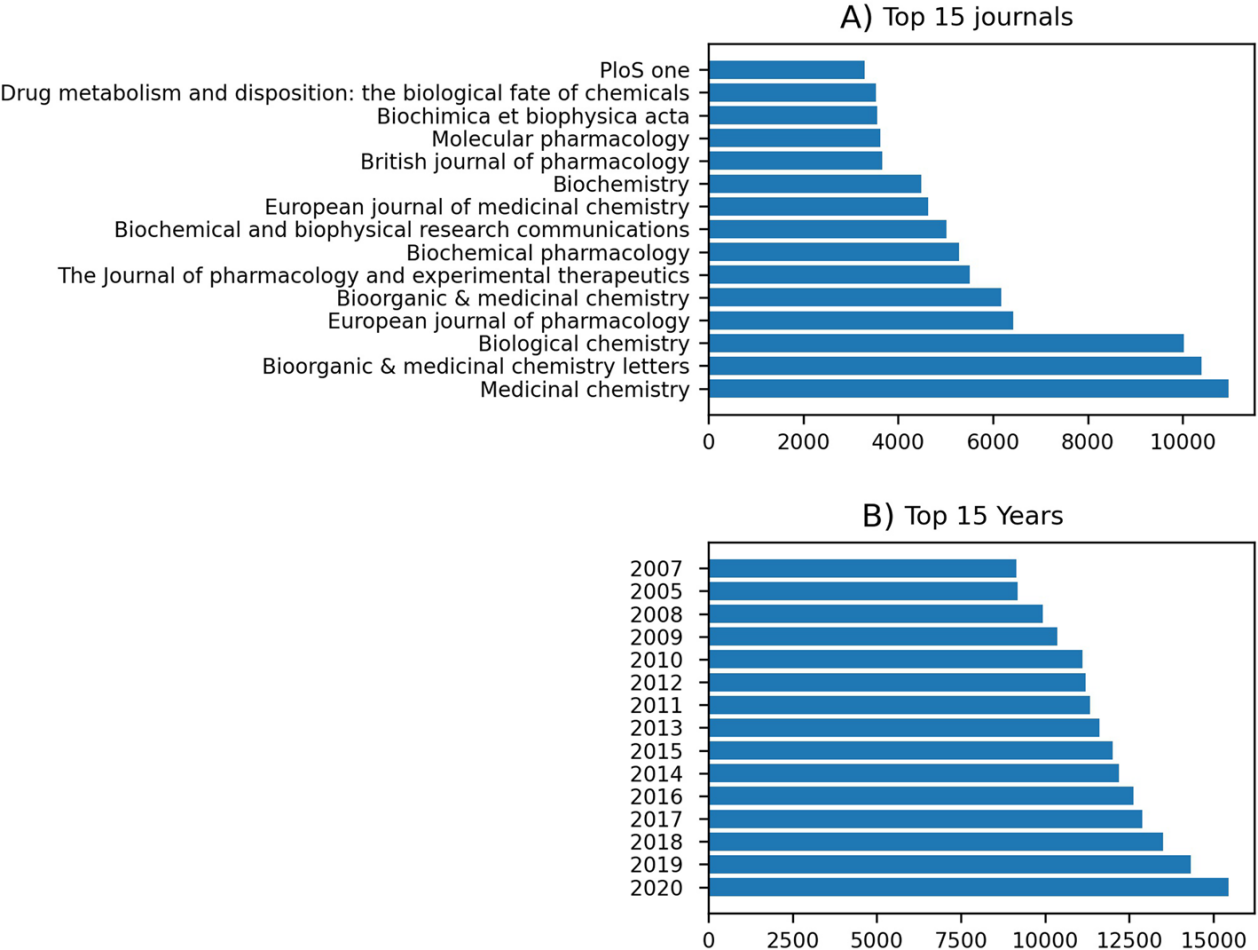
**A** Top 15 journals for the articles that are predicted as drug-target based articles, **B** Top 15 years for articles predicted as drug-target articles

We also analyzed the publication journals and years for these predicted articles. We found that Journal of Medicinal Chemistry, Bioorganic & Medicinal Chemistry Letters and Biological Chemistry are the top three journals based on our prediction (Fig. 4A). These three journals are also among the leading journals for bioactivity data extraction in ChEMBL [39]. Furthermore, most drug-target articles are from recent years, with the year of 2020 containing the most significant number of articles (Fig. 4B).

The output of our analysis can be used as a starting point to further extract the quantitative drug-target bioactivity values from the identified articles. We hope that our output will significantly ease the job of manual curators as we are providing the actual PubMed ID, drugs, and protein entities, as well as assay formats for newly identified DTI articles.

### Predict assay format for drug-target articles

After successfully identifying DTI articles, the next task is to predict the assay format that was reported. For that purpose, we separately trained each BERT model with 14,109 articles based on cell-based assay, 12,845 articles based on organism-based assay, and 1148 with the other assay formats. We used the same fine-tuning settings as for the drug-target article identification task and used F1 macro and F1 micro metrics to evaluate the performance of the models. We observed better accuracy improvements when using the weighted cross entropy (each class with a different weight based on effective number of samples) defined as:

$${\text{WCE }}\left( {{\text{y}},{\tilde{\text{y}}}} \right) = { } - \mathop \sum \limits_{n}^{{\text{N}}} {\text{w}}_{{y_{n} }} . {\text{y}}_{n} {\text{ log }}\left( {{\tilde{\text{y}}}_{n} } \right){ }$$

where $y$ is the target and $w$ is the weight and the predicted class label $\stackrel{\sim }{\mathrm{y}}$ is the index of the maximum predicted probability score among the three classes.

Table 3 shows tenfold cross-validation performances for the BERT models. We found that BioMed-RoBERTa outperforms the other models, with F1 micro of 88.1 ± 0.5, and F1 macro of 87.8 ± 0.5. The superior performance of BioMed-RoBERTa could be due to the additional pre-training over more data consisting of 2.68 M full-text papers from S2ORC [41] and the additional pre-training for longer steps.

**Table 3:** The tenfold cross validation results for identifying assay formats

BERT model	F1 macro	F1 micro
BERT	81.0 ± 1	81.5 ± 1
SciBERT	85.2 ± 1.1	85.6 ± 1.1
BioBERT	86 ± 1.3	86.4 ± 1.3
BioMed-RoBERTa	**87.8 ± 0.5**	**88.1 ± 0.5**
BlueBERT	87.0 ± 1	87.5 ± 1

Bold values indicate the top results for a dataset

After successful finetuning of BERT models to predict assay formats (of known articles), we used the best model (i.e., BioMed-RoBERTa) to predict assay formats for 597,844 articles (identified as drug-target articles using majority voting in Table 2). BioMed-RoBERTa predicted that 243,828 (out of 597,844) articles are cell based, 220,357 articles are organism-based articles, and 133,659 articles are others assays.

## Discussions and conclusions

More than 80% of the approved drugs target only two protein classes: enzymes or receptors [42]. There are 25 000 genes in humans, but only 600 disease-modifying protein drug targets exist [43]. Therefore, target identification has recently shifted to other macromolecules, such as RNAs. Due to their involvement in gene regulation, miRNAs have been identified as high-value targets for therapy. There are approximately 2000 miRNAs in humans (www.mirbase.org). They regulate 30% of all genes which are crucial in many biological processes [44, 44]. Therefore, traditionally ‘undruggable’ proteins can be targeted via their miRNA gene regulators, enabling the treatment of incurable diseases [46]. Recently in-silico methods have been developed to predict drugs for miRNA. For instance, Chen et al. proposed a bounded nuclear norm regularization method [47]. Niu et al. adapted graph neural network-based method to predict drug resistance for miRNAs [48]. Several more methods are published on in-silico drug associations with miRNA [49–51].

However, in this study, our focus is mainly on protein targets due to (1) insufficient miRNA targets available in the public databases and ‘(2) Lack of miRNAs annotations at PubTator [28], which is the main source of our pipeline. Therefore, we omitted miRNAs and focused only on protein targets in the present study. However due to the growing importance of miRNAs as emerging drug targets, in future, we aim to also include miRNA in text-mining based drug-target relationship extraction and combine it with machine learning-based prediction method to identify novel drug associations with miRNA.

In target centric drug discovery, a large number of compounds are tested across a particular target protein, resulting in the lack of DTI profiles at the proteomics level for many compounds. Curating quantitative drug-target bioactivity values reported in an article is therefore a critical task for establishing a more comprehensive drug-target profiles. Semi-automated NLP based methods can assist in identifying such articles and easing the workload for the data curators. BERT is recently proposed as a state-of-the-art model for several NLP tasks, including text classification. Therefore, in this research, we investigated several models of BERT to identify new articles likely containing DTIs.

Furthermore, we developed these models to predict the assay formats most likely used in the articles. Assays formats are critical in evaluating the quality for DTIs. We found that BioMed-RoBERTa performed slightly better than the other models for both drug-target article identification and assay format prediction.

Using the majority voting based on BERT models, we identified 597,844 articles from which 316,794 are confirmed to have both drug and protein entities in PubTator. Most of these articles are not reported in any of the manually curated bioactivity databases as the combined non-overlapping articles curated by commonly used DTI databases are around 0.1 M. These identified DTIs (along with annotations) are freely available at https://dataset.drugtargetcommons.org/. We hope that the identified articles and drug and protein entities will ease the job of manual curators and improve protein target coverage across investigational and approved compounds. Lastly, increased target coverage for investigational and approved drugs will enhance the understanding of drug mechanism of action and open new drug repurposing opportunities. The manual curation team of DrugTargetCommons will take advantage of these newly identified articles and curate bioactivity data. Meanwhile, we will try to extend our recently published method on drug target relationship extraction [52] to automatically identify DTI relationships from these articles.

## Supplementary Information

**Additional file 1**. Pmids for 100 articles that are manually validated to contain drug-target interactions.

## Data Availability

Newly identified articles, extracted drug/protein entities and predicted assay formats are freely available at https://dataset.drugtargetcommons.org/.
